# Deposition of Mesoporous Silicon Dioxide Films Using Microwave PECVD

**DOI:** 10.3390/ma18133205

**Published:** 2025-07-07

**Authors:** Marcel Laux, Ralf Dreher, Rudolf Emmerich, Frank Henning

**Affiliations:** 1 Fraunhofer Institute for Chemical Technology, Joseph-von-Fraunhofer-Straße 7, 76327 Pfinztal, Germany; 2 Karlsruhe Institute of Technology, Institute of Vehicle System Technology, Rintheimer Querallee 2, 76131 Karlsruhe, Germany

**Keywords:** mesoporous, microwave, PECVD, silica, SiO_2_, silicon dioxide, hexamethyldisiloxane, adhesion layer, polymer–metal, injection molding

## Abstract

Mesoporous silicon dioxide films have been shown to be well suited as adhesion-promoting interlayers for generating high-strength polymer–metal interfaces. These films can be fabricated via microwave plasma-enhanced chemical vapor deposition using the precursor hexamethyldisiloxane and oxygen as working gas. The resulting mesoporous structures enable polymer infiltration during overmolding, which leads to a nanoscale form-locking mechanism after solidification. This mechanism allows for efficient stress transfer across the interface and makes the resulting adhesion highly dependent on the morphology of the deposited film. To gain a deeper understanding of the underlying deposition mechanisms and improve process stability, this work investigates the growth behavior of mesoporous silica films using a multiple regression analysis approach. The seven process parameters coating time, distance, chamber pressure, substrate temperature, flow rate, plasma pulse duration, and pause-to-pulse ratio were systematically varied within a Design of Experiments framework. The resulting films were characterized by their free surface area, mean agglomerate diameter, and film thickness using digital image analysis, white light interferometry, and atomic force microscopy. The deposited films exhibit a wide range of morphological appearances, ranging from quasi-dense to dust-like structures. As part of this research, the free surface area varied from 15 to 55 percent, the mean agglomerate diameter from 17 to 126 nm, and the film thickness from 35 to 1600 nm. The derived growth model describes the deposition process with high statistical accuracy. Furthermore, all coatings were overmolded via injection molding and subjected to mechanical testing, allowing a direct correlation between film morphology and their performance as adhesion-promoting interlayers.

## 1. Introduction

Mesoporous silica thin films exhibit a variety of promising properties. They have been utilized in optical applications as anti-reflective [1,2,3,4] or anti-fogging coatings [5], in medical applications as bioactive materials [6,7], in drug delivery systems [8,9], and as a platform for thin-film sensors [10,11]. Mesoporous structures can be generated through a range of methods, including dip-coating [11,12], sol-gel processing [6,13], spin-coating [7], layer-by-layer assembly [5,14], or a combination of spin-coating and downstream plasma treatment [15]. In 2011, Emmerich et al. [16] first demonstrated that mesoporous silica thin films could also serve as adhesion-promoting interlayers to create high-strength polymer-metal interfaces. These mesoporous films were deposited using microwave plasma-enhanced chemical vapor deposition (mPECVD) with hexamethyldisiloxane (HMDSO) and oxygen as precursors. Microwave PECVD offers several advantages for the deposition of silica-based thin films. The high electron density of microwave-driven low-pressure plasmas enables elevated deposition rates compared to other plasma-enhanced processes [17,18,19,20]. At the same time, the process operates under so-called “cold plasma” conditions, ensuring precise thermal control and allowing coating at low substrate temperatures, which is essential for processing temperature-sensitive materials such as polymers. The use of HMDSO as a silicon-containing precursor further contributes to the suitability of the method. HMDSO is characterized by a high vapor pressure, low toxicity, and favorable reactivity in oxygen-containing plasmas [21]. Together, these characteristics make mPECVD a highly efficient and versatile technique for the low-temperature deposition of mesoporous silica thin films on a broad range of substrate materials.

Emmerich et al. [16] postulated that the adhesion mechanism is based on a nano-interlocking effect between the mesoporous SiO_2_ structure and the infiltrating polymer. Follow-up studies by Laux et al. [22] confirmed these results and demonstrated that such mesoporous adhesion layers are suitable for thermoplastic injection molding. However, significant scatter in the measured adhesion values was observed. Laux et al. attributed these variations in interfacial strength to inconsistent infiltration of the mesoporous films and suggested that the process window for sufficient infiltration could be extended by tailoring the film morphology to the specific thermoplastic polymer [23].

The present study therefore investigates the extent to which the morphology of mesoporous SiO_2_ films can be tuned by adjusting deposition parameters. The films were characterized using digital image analysis, then overmolded via injection molding and subjected to mechanical testing, enabling a direct correlation between their morphology and their performance as adhesion-promoting interlayers.This approach opens up the possibility to identify previously unexplored mesoporous structures and to investigate the influence of film morphology on the interfacial strength of injection-molded SiO_2_-polymer systems in greater detail. Ultimately, this may lead to the development of films with enhanced mechanical strength that allow for more reliable and easier infiltration during injection molding or comparable processes, which is an requirement for the industrial implementation of such mesoporous adhesion films.

## 2. Materials and Methods

In order to describe the growth behavior of the mesoporous silicon dioxide-based thin films, a total of seven deposition parameters were varied in a statistically planned parameter study as can be seen in Table 1. The effect on film growth behavior was evaluated based on specifically defined film characteristics such as free surface area (FSA), mean agglomerate diameter (MAD), and film thickness (*h*), which are described in more detail in Section 2.3.

### 2.1. Deposition of the Mesoporous Silicon Dioxide Films

**Figure 1:**
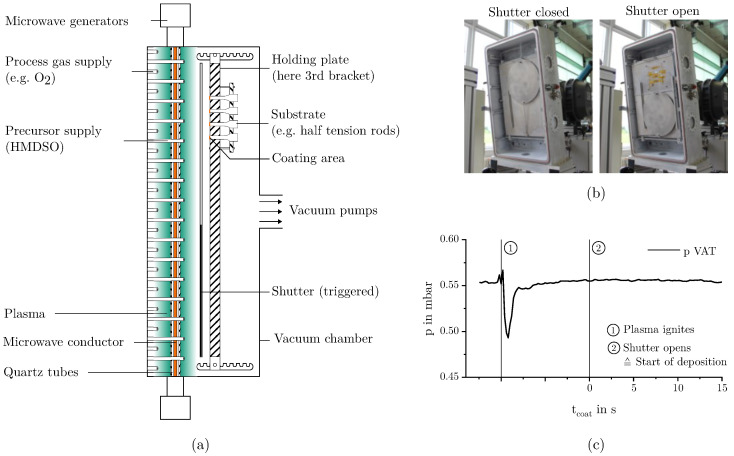
(**a**) Schematic sketch of the coating array; (**b**) deposition area with shutter closed (left) and opened (right); (**c**) qualitative pressure curve during deposition.

All films investigated were deposited on an experimental plant, based on the plasma-duoline principle at the Fraunhofer Institute for Chemical Technology (ICT). The coating array configuration is shown schematically in Figure 1a. The vacuum chamber measures 1000 mm in height, 560 mm in width, and 337 mm in depth. It is equipped with 144 gas feed tubes that supply the precursor molecule HMDSO. These are arranged in such a way that the quartz tubes are not unnecessarily coated during deposition. In addition, there are 128 gas feed tubes that supply the process gases. The arrangement of all tubes ensures a homogeneous distribution of both the precursor and the process gases across the entire coating surface. The pumping station consists of three vacuum pumps connected in series. The first vacuum pump is a screw line pump (Model: SP250 from Leybold, Germany) with an individual throughput of 250 m^3^/h. The second vacuum pump is a roots pump (Model: WA1001 11740 from RUVAC, Germany), which delivers a volume flow rate of 1000 m^3^/h. The third vacuum pump is also a roots pump (Model: 900615MHR601 from Edwards s.r.o., Czech Republic), delivering a maximum volume flow rate of 3500 m^3^/h. The pumping station is connected via an DN 200 recipient regarding ISO 3669:2020 [24] and enabled an actual throughput of around 2800 m^3^/h at 5–30 standard liter per minute (SLPM) and around 2000 m^3^/h at gas flow rates of less than 5 SLPM. Aluminum pin stubs with a diameter of 12.5 mm (Plano GmbH, Wetzlar, Germany) and microscopy glass slides compliant with ISO 8037/1 [25] (Epredia, Portsmouth, NH, USA) were used as substrate materials for the following characterization. The aluminum pin stubs were mechanically and chemically polished to minimize the influence of surface roughness on the following image evaluation. All specimens were carefully cleaned and then mounted on an aluminum retaining plate as can be seen in the left picture of Figure 1b. The retaining plate has dimensions (height × width) of 690×520 mm and was placed centrally within the chamber. The aluminum pin stubs had a diameter of 13 mm and were located 100 mm below the upper edge of the holding plate and 130 mm from its right side edge. Pin stubs were flush mounted on the retaining plate and screwed from behind to prevent edge effects. The glass slides were mounted directly underneath and partially covered with polyimide adhesive tape. In the following, the distance between the gas showers that distribute the precursor HMDSO and the substrate surface is referred to as distance *dis*. The circular aluminum shutter had a diameter of 400 mm and was placed in the center line of the vacuum chamber. The shutter could be activated from outside the chamber, causing it to fall down and release the samples.

Before the actual coating process, a pre-treatment with an opened shutter was performed. For both the pre-treatment and the actual coating process, the array was respectively pumped down from atmospheric pressure to a process start pressure or residual gas content of less than 0.02 mbar, which minimized the risk of disturbing influences caused by residual gases and therefore increased reproducibility. Once the pressure had reached its target value, the substrates were cleaned and activated with oxygen plasma for 20 s with an oxygen flow of 2 SLM, as the surface activation provides better adhesion at the substrate–SiO_2_ interface [26,27]. After pre-treatment the vacuum chamber was vented and the shutter was positioned in front of the samples.

Subsequently, the chamber was evacuated to below 0.02 mbar again. The samples were then heated slightly above their desired target substrate temperature $T_{Sub}$ using a hydrogen plasma. Gas flows were 2 SLM of hydrogen + 0.2 SLM of argon for plasma homogenization. To initiate the actual coating process, the gas mixture was switched from the pre-treatment gas to the coating gas mixture, consisting of HMDSO and oxygen. Once the pressure and the gas flow conditions stabilized and the substrates were cooled down to their exact target temperature $T_{\mathrm{Sub}}$, the plasma was ignited by activating the microwave power. As the formation of the plasma causes a temporary increase in chamber pressure *p*, which is counter-regulated by the proportional control valve, a more or less pronounced pressure drop occurs in the first few seconds of the coating due to excessive control response, as can be seen in Figure 1c. Since an undefined deposition would occur during this time, the substrates were covered by a shutter as shown in Figure 1b. After reaching constant deposition conditions, the shutter releases the substrates, and the actual deposition process starts ($t_{\mathrm{coat}}=0$ s). The reactants HMDSO and oxygen react by plasma energy and form silicon dioxide, carbon dioxide, and water as reaction products. The chemical conversion takes place according to the following conversion equation:

(1) $${\left({\mathrm{CH}}_{3}\right)}_{3}\mathrm{Si}\text{−}O\text{−}\mathrm{Si}{\left({\mathrm{CH}}_{3}\right)}_{3}+12\;O_{2}\overset{\mathrm{Plasma}\;\mathrm{energy}}{\to }2\;{\mathrm{SiO}}_{2}+6\;{\mathrm{CO}}_{2}+9\;H_{2}O$$

Depending on the deposition parameters, the main product silicon dioxide is deposited as a mesoporous film. Both side products, carbon dioxide and water, are sucked through the vacuum chamber venting valve. The deposition was performed with a slightly hyperstoichiometric ratio which was reached by adding 12.7 parts of oxygen to one part of HMDSO (ratio = 12.7:1). This ensured that a relatively pure SiO_2_ film was deposited without carbon impurities. The precursor vapor was fed in pure form, without carrier gas. A mass flow controller regulates the gas flows at a defined ratio. The total flow of the precursor HMDSO is being described by the flow rate *Q* which is specified in standard cubic centimeters per minute (SCCM). After each adjustment of the HMDSO flow rate, the amount of oxygen added was aligned according to the constant ratio of 12.7:1. Unregulated, the inflow of the reactants would typically lead to chamber pressures of around 0.1 to 0.4 mbar. Therefore, a proportional–integral–derivative (PID)-controlled throttle valve raises and stabilizes the chamber pressure *p* at its target values at the fixed process gas flows. Throughout the whole study all eight microwave generators operated at their maximum power of 4 kW per generator and the applied microwave power was adjusted by the parameters pulse duration $t_{\mathrm{pulse}}$ and pause-to-pulse ratio *PtPR*. The lower the *PtPR*, the higher the microwave power applied. The microwave power correlates directly with the applied plasma power. The reason for always operating with pulses and using a maximum pulse power was to ensure the most homogeneous plasma discharge, thereby providing a uniform plasma over the entire area. This is especially relevant at higher pressures and considering that no argon was mixed into the gas during the coatings.

### 2.2. Parameter Selection and Experimental Design

Preliminary screening experiments had shown that all seven investigated process parameters have a significant influence on film growth. However, including this number of variables results in a high level of experimental complexity. Therefore, an empirical approach based on a Design of Experiments (DoE) methodology was deliberately chosen. This strategy allows for the identification and modeling of relationships between the process parameters and the resulting film properties through regression analysis, without requiring a full theoretical description of the underlying physical and chemical mechanisms. Nonetheless, theoretical considerations were applied selectively to verify the plausibility of the regression model outputs and to support the interpretation of observed trends. The DoE followed a three-level face-centered central composite design (CCF). The three-parameter level design enables the determination of non-linear and quadratic interactions while significantly reducing the experimental effort compared to a full factorial design [28]. The statistical modeling was conducted using the data analytic software MODDE 12 from Sartorius, Germany.

The parameter boundaries were deliberately chosen to ensure mesoporous film growth throughout the entire experimental design space. This decision was made to avoid fundamental and potentially abrupt transitions in growth behavior, which could negatively impact the quality of the resulting regression models, as discontinuities are difficult to capture accurately using the mathematical modeling approaches applied in this study. The individual parameter levels are shown in Table 1.

The coating time was varied between 5 and 25 s. The lower limit was chosen to ensure that a porous (though thin) film could still form even with low deposition rates. The upper limit was based on expected deposition rates and previous results showing that film thicknesses from depositions longer than 25 s often lead to unwanted densification or cracking, which would reduce adhesion quality.

The distance was set based on the minimum and maximum values that can be technically realized in the coating system, whereas the center point of the chamber pressure was selected based on previous work by Emmerich et al., and the pressure range was limited to values where the array could still generate a stable and uniform plasma.

The precursor flow rate was chosen to maintain the desired pressure reliably across all parameter combinations without overloading the vacuum system.

The substrate temperature ranged from room temperature to 110 °C, and was selected based on earlier studies showing that mesoporous films form best at lower temperatures when using microwave PECVD. The targeted film morphology in this study was intentionally porous, as the adhesion mechanism under investigation relies on nanoscale mechanical interlocking. This mechanism requires sufficient surface porosity, which is favored at lower substrate temperatures. Therefore the process conditions were also selected to remain within Zone 1 of the structure zone model. The chosen upper limit of the substrate temperature (110 °C) corresponds well with the transition from Zone 1 to Zone 2, which typically occurs at approximately 20% of the melting temperature of SiO_2_ [29]. Furthermore, the relatively low substrate temperature investigated was consistent with one of the core advantages of mPECVD, which is its ability to operate at low thermal budgets due to the intrinsically cold nature of microwave-excited plasmas.

The plasma power was applied in a pulsed mode to deliberately exploit the so-called afterglow effect. This allows the plasma to operate at a reduced average energy input, resulting in lower thermal load while still maintaining a sufficiently high electron density during the afterglow phase [21,30,31]. By adjusting both the pause-to-pulse ratio and the pulse duration, a stable and homogeneous plasma operation could be ensured. Continuous operation or operation at too low microwave power would have led to spatially inhomogeneous plasma conditions and compromised process stability. The upper limit of the pulse frequency was set between the technical maximum of the system and the lowest value, which still produced an apparent afterglow effect.

A multiple linear regression (MLR) approach was adopted to model the responses. All models were fitted using an augmented backward elimination approach [32,33,34]. Initially, a comprehensive model encompassing all main effects, first-order interactions, and quadratic interactions was developed. Subsequently, non-significant model terms, identified through *p*-value analysis using F-tests, were step-wise eliminated from the model equation. After each removal, the regression model was recalculated and the quality of the model was assessed based on changes in key metrics such as the coefficient of determination (R^2^), the predictive relevance (Q^2^), and the model validity [35]. The objective was to achieve a model that effectively captured the relationship between input and output variables without overfitting. This process was repeated until no model term with a *p*-value greater than 0.05 remained in the model equation, or until further simplification led to a decrease in model performance as indicated by a drop in Q^2^.

### 2.3. Characterization of the Deposited Film Morphology

In order to analyze the deposited films for modeling precisely, the morphology was characterized by the parameters free surface area (FSA), mean agglomerate diameter (MAD), and film thickness *h*. The morphology of the deposited layers was characterized using automated image evaluation based on scanning electron microscopy (SEM) images. To obtain sharp images with high contrast and to prevent electric charging, it was necessary to sputter all samples for 20 s with gold–palladium (60 gold/40 palladium) before taking the SEM pictures. The sputtering was conducted under an argon atmosphere at 0.08 mbar chamber pressure and a current of 40 mA using a 208HR sputter coater from Cressington Scientific Instruments, Watford, UK. All images were taken with a Supra 55 VP SEM from ZEISS, Oberkochen, Germany. The exposure parameters were defined based on previously performed manual measurements of the agglomerate sizes. A complete analysis of the expected particle sizes required a magnification factor of twenty thousand at an image resolution of 1024 × 768 pixels. The operating distance was set to 3 mm, and the chamber pressure was set to values between 1 and 3 mbar. In order to display only the surface topography of the deposited layers, a secondary electron detector (SED) was used with a reasonably low acceleration voltage of 1 kV, according to the work of Zarraoa et al. [36] and Joy [37].

**Figure 2:**
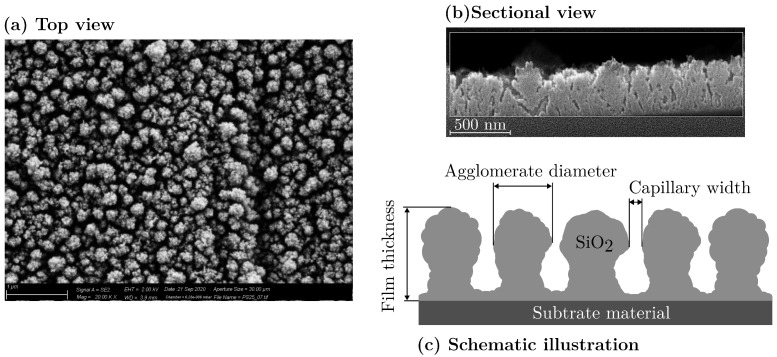
(**a**) Top view SEM image of the mesoporous SiO_2_ film at a magnification level of twenty thousand; (**b**) sectional view SEM image of the SiO_2_ film made by ion beam cutting; (**c**) schematic illustration of the film morphology.

The deposited films are composed of many individual agglomerates that grow together, building a column-like mesoporous structure as shown in Figure 2a. Following the definition of the International Union of Pure and Applied Chemistry, the pores will be referred to as mesoporous in the course of this work [38]. Depending on the specific deposition conditions, the layer morphology ranged from quasi-dense structures with the smallest agglomerates of a few nanometers to more or less dust-like layers with agglomerate diameters of over 120 nanometers. An ion beam cutter was used to expose a side view of the layer structure at a magnification of one hundred thousand times (see Figure 2b). It can be seen that the agglomerates grow like columns with an upward-increasing diameters. The forming structures are schematically sketched in Figure 2c. While growing, the columnar SiO_2_ agglomerates form more or less pronounced cavities that can be described as capillaries. This growth behavior has already been shown by Emmerich et al. [16]. It is assumed that particle flow effects are responsible for the upwards-increasing diameter. Neither closed nor dust-like layers are suitable as adhesion layers for polymer–metal hybrids [23]. Therefore, this work aims to describe the parameter space between these film characteristics as accurately as possible.

#### 2.3.1. Free Surface Area

To determine the FSA, scanning electron microscope images of the SiO_2_-coated pin stubs were made and binarized using automated gray-level threshold image segmentation. For the FSA, a local adaptive binarization method, according to Sauvola and Pietikäinen [39] was employed. The Sauvola threshold method further develops Niblacks local threshold method [40] with the primary goal of improved noise performance. The advantages of local threshold methods are that the threshold value is continuously recalculated and adjusted depending on the locally prevailing brightness and contrast conditions. The threshold method, according to Sauvola and Pietikäinen, thus guaranteed a robust detection of the capillaries and showed to be very robust against local brightness as well as contrast differences [41].

As shown in Equation (2) the threshold for a pixel T(x,y) is calculated from the mean value m(x,y) and the standard deviation s(x,y) of the gray scale g(x,y) of a N×N large pixel environment according to Equation (2).

(2) $$\begin{array}{c} T\left(x,y\right)=m\left(x,y\right)\left[1+k(\frac{s(x,y)}{R}-1)\right] \end{array}$$

The mean value m(x,y) and the standard deviation s(x,y) are determined as follows:

(3) $$\begin{array}{c} m\left(x,y\right)=\frac{1}{N^{2}}\sum_{i=-\frac{N-1}{2}}^{\frac{N-1}{2}}\sum_{i=-\frac{N-1}{2}}^{\frac{N-1}{2}}g(x+i,y+i) \end{array}$$

(4) $$\begin{array}{c} s\left(x,y\right)=\sqrt{\frac{1}{N^{2}}\sum_{i=-\frac{N-1}{2}}^{\frac{N-1}{2}}\sum_{i=-\frac{N-1}{2}}^{\frac{N-1}{2}}{\left[g(x+i,y+i)-m(x,y)\right]}^{2}} \end{array}$$

**Figure 3:**
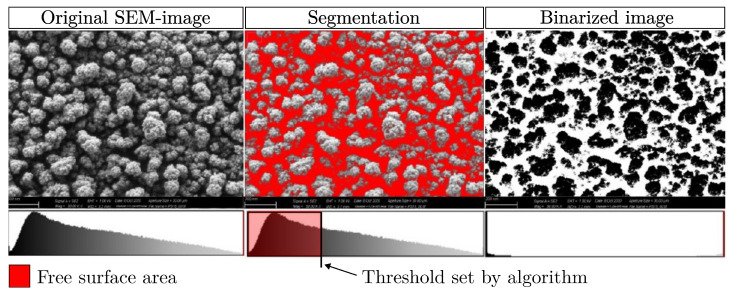
Approach to automated image segmentation using the Sauvola algorithm as an example.

*R* is the dynamic range of standard deviation and is set to the default value of R=128, as recommended in the original Sauvola publication. The size or radius of the evaluation window to compute s(x,y) and m(x,y), centered on the current pixel, is called *N*. In this investigation *N* was set to N=15. To ensure that only capillaries and no pre-existing changes in surface roughness are detected, it was necessary to prevent the algorithm from becoming too sensitive. Following the investigations of Lazzara et al. [42], this could be achieved by setting the Niblack constant *k* to a relatively high value of k=0.5, so that only sharp changes in the gray level are detected. The free surface area is defined by the proportion of area obtained after segmentation and binarization (see Figure 3) and corresponds to the percentage of near-surface capillaries related to the total surface area of the sample.

#### 2.3.2. Mean Agglomerate Diameter

To accurately determine the MAD, a different segmentation algorithm was required. While the Sauvola algorithm effectively identifies capillary areas, it falls short in adequately separating individual agglomerates. Agglomerates identified using the Sauvola algorithm often appear connected at thin points, resulting in their detection as a single cohesive structure. Consequently, this leads to an overestimation of agglomerate size and an underestimation of the total number of agglomerates. However, precise delineation is essential for accurately measuring and enumerating individual agglomerate segments, which is critical for calculating the MAD.

In contrast, the MaxEntropy algorithm, specifically optimized for segmentation tasks, yields significantly improved results [43]. This threshold-based method determines the threshold value based on the entropy of the histogram. Unlike the Sauvola method, the MaxEntropy approach tends to overestimate the proportion of capillaries. Nevertheless, post-binarization, it provides better-defined agglomerate segments, enhancing their distinction from one another. The agglomerates are then counted, and their respective areas are calculated in square pixels. A lower pixel threshold of 60 pixels was set to detect agglomerates. Additionally, to mitigate potential distortions from local poor exposure conditions or layer defects during evaluation, agglomerates larger than 5000 square pixels were also excluded from analysis.

For better comparability, the mean agglomerate size in square pixels was converted to a circular diameter using Equation (5). A conversion factor of 0.18 facilitates the transition from square pixel to square nanometers at a magnification of 20,000 and a resolution of 1046×768 pixels.

(5) $$\begin{array}{c} \mathrm{MAD}\;\left[nm\right]=\sqrt{\frac{4\cdot \mathrm{MAD}\;\left[\mathrm{square}\;\mathrm{pixel}\right]}{0.18\cdot \pi }} \end{array}$$

It is important to note that the characterization methods described are limited to evaluating areas close to the surface and that it can be assumed that both the FSA and the MAD vary with film thickness. In addition, although the FSA could be interpreted as near-surface film porosity, porosity is technically defined as the ratio of cavity volume to the total volume of a substance [44]. Since this work employs a two-dimensional evaluation method, the standard definition of porosity was intentionally not applied.

#### 2.3.3. Film Thickness

**Figure 4:**
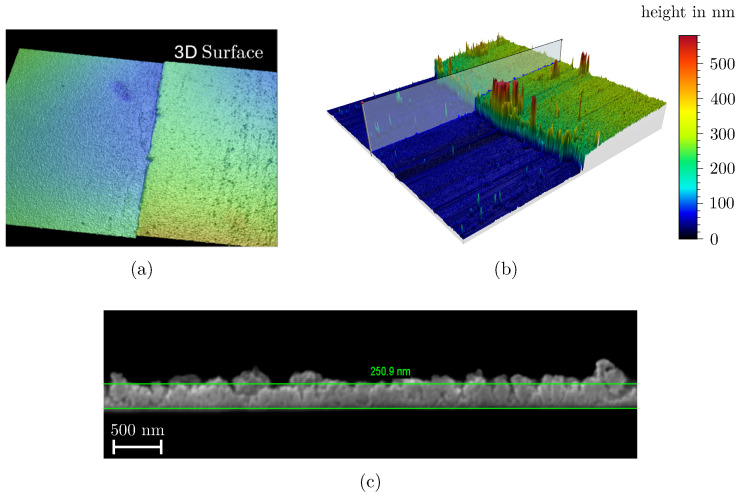
Overview of the measurement methods used to determine the film thickness of the deposited mesoporous films. (**a**) White light interferometry; (**b**) atomic force microscopy; (**c**) ion beam sections for subsequent measurement in SEM.

The film thickness *h* was determined on a NT 1100 Optical 3D Surface Profiler (Veeco Instruments Inc., Plainview, NY, USA) using white light interferometry. For this purpose, the microscopy glass slides were partially masked with polyimide adhesive tape during the coating process. After removing the adhesive tape, the samples were sputtered with a 40 nm thick gold–palladium film, which served as a reflective layer for the white light interferometry. Measurements were taken at three locations and then averaged. The substrate surface characterized by white light interferometry is shown in Figure 4a. To validate the accuracy of the measurements, selected data points were randomly cross-checked using atomic force microscopy (AFM), as illustrated in Figure 4b. Additionally, ion beam cross-sections were prepared for some of the films and compared with the white light interferometry results, as shown in Figure 4c. The deviation between the measurement methods was within the standard deviation of the mean value formation observed with the white light interferometry. Therefore, it was assumed that the thickness measurement using white light interferometry provides valid results, as AFM measurements and ion beam sections could only be carried out with some samples due to the high effort required.

#### 2.3.4. Mechanical Testing of the Interfacial Tensile Strength

**Figure 5:**
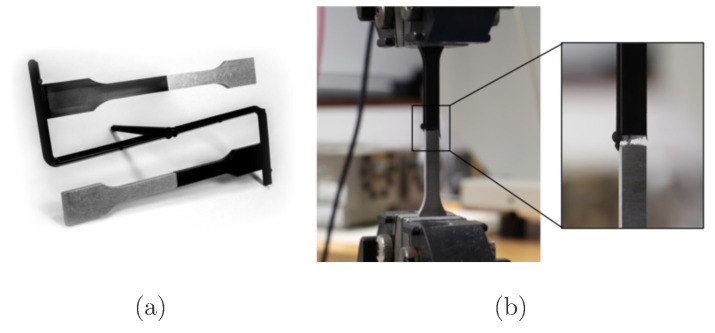
(**a**) Hybrid test specimens after demolding; (**b**) mechanical characterization according to ISO 6892 [46].

To establish a direct correlation between the coating parameters and the adhesive tensile strengths of the injection-molded polymer–SiO_2_ interface, all coatings produced within the film growth DoE were additionally characterized mechanically using hybrid polymer–metal tensile bars, as shown in Figure 5a. For each coating cycle, ten half-standard tensile specimens made of aluminum were positioned in the array and coated on their frontal sides. For this, proportional flat specimens were cut from the rolled alloy EN AW 5754 [45] with a sheet thickness of 4 mm using water jet cutting. The cutting parameters were adjusted so that the angled edge caused by the water jet corresponded to the draft angle of approximately 3 degrees in the mold insert, ensuring secure demolding and preventing lateral overmolding. To minimize the influence of surface roughness on the adhesive tensile strength and to measure only the pure effect of the mesoporous SiO_2_-film, the frontal faces of the half-tensile specimens were polished flat in three steps using wet grinding paper (grit sizes P800, P1200, and P2400) on a grinding and polishing machine LaboPol 30 from Struers S.A.S., Courtabœuf, France. The samples were centered in a clamping tool in groups of ten to ensure flatness and eliminate transverse forces during subsequent tensile testing. After grinding, the samples were cleaned with isopropanol and mounted flat with their frontal side to the holding plate of the coating array.

The hybrid tensile bars were then overmolded on their frontal side using an Engel Victory 330/120 injection molding machine (Engel Austria GmbH, Schwertberg, Austria) with the polyphenylene sulfide (PPS) compound Xytron G3010E (DSM Engineering Materials, Geleen, The Netherlands) as shown in Figure 5a. The G3010E is reinforced with 30 percent glass fibers and is characterized by high strength, high impact resistance, dimensional stability, and stiffness. Due to its low melt viscosity, it was assumed that even mesoporous films that are more difficult to infiltrate could be penetrated. Furthermore, its high strength was expected to enable mixed-mode failure in even high-strength SiO_2_ films, thereby allowing the identification of potential weak points within the film structure. The PPS was pre-dried for five hours at 120°C and the injection molding parameters were set as shown in Table 2. The tension bar mold was equipped with an ejector bar, which evenly distribute the ejector force along the length of the specimens and prevents damage to the hybrid tensile specimens during demolding.

The hybrid tensile specimens were tested according to ISO 6892 [46], using a tensile testing machine Inspekt Table 50 from Hegewald & Peschke from Nossen, Germany, at a speed of 1 mm/min until failure as shown in Figure 5b. The tests were conducted under the laboratory environment at room temperature (23 ± 3 °C and 50 ± 5% relative humidity). All specimens failed as planned at the interface between aluminum, SiO_2_, and PPS. The adhesive tensile strength in MPa of the interface was calculated based on the maximum measured breaking force F_max_ in Newton and the nominal cross-section (A = 40 mm^2^, with 10 mm width and 4 mm height) regarding equation:

(6) $$\begin{array}{c} \sigma =\frac{F_{max}}{A} \end{array}$$

## 3. Results

First, a qualitative overview of the observed coating morphology is given, followed by a more detailed discussion of the regression models derived for the responses MAD, FSA, and film thickness.

**Figure 6:**
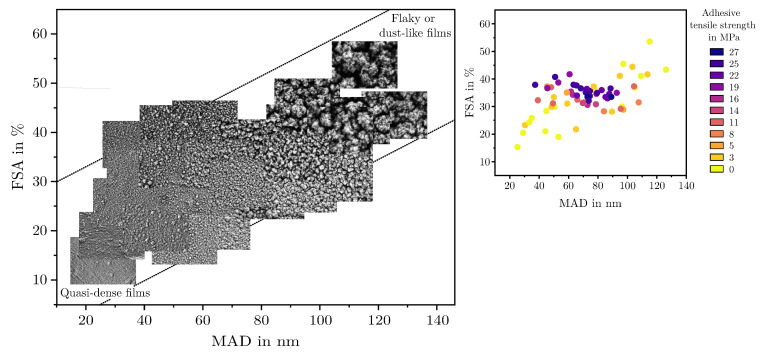
Overview of the films deposited within the DoE, sorted by their MAD and FSA, along with the corresponding measured adhesion tensile strengths after overmolding with a 30 wt% glass fiber reinforced PPS.

Based on the characterization methodology defined in Section 2.3, mesoporous films with an FSA of 15 to 55 percent, a MAD of 17 to 126 nm, and a film thickness of 35 to 1600 nm were observed within the DoE. Figure 6 shows scanning electron microscope images of the films deposited, sorted according to their FSA and MAD, which serve as key descriptors of their nanostructure. The measured adhesion tensile strengths to a 30 wt% glass fiber-reinforced PPS are also shown in the right part of the diagram, which allows a direct comparison of structure–property relationships and highlights the influence of film morphology on the resulting hybrid joint strength. Films that were marked as outliers in the model fitting are not included in the composition. This particularly affected extremely dusty layers (high pressure, high flow rate, large distance, and low temperature) or very thin films (low pressure, low flow rate, small distance, and short coating times).

**Figure 7:**
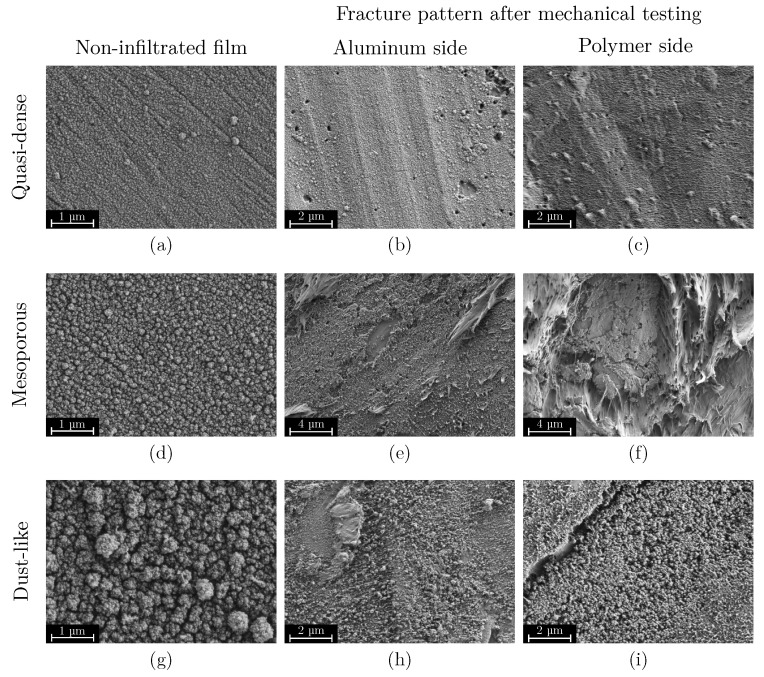
Overview of characteristic mesoporous film structures prior to overmolding (**left**) and the corresponding fracture surfaces on the substrate side (**center**) and the polymer side (**right**). (**a**–**c**) The first row shows a quasi-dense film structure, located in the transition region between a quasi-dense and mesoporous morphology, with a film surface area (FSA) of 29% and a mean absolute deviation (MAD) of 60 nm. This film did not show adhesion tensile strength in mechanical testing. (**d**–**f**) The second row represents a mesoporous film structure with an FSA of 33% and a MAD of 80 nm. The film generated a strong interfacial bond with the polymer, resulting in a tensile strength of up to 30 MPa. (**g**–**i**) The third row shows a dust-like film morphology with an FSA of 37% and a significantly higher MAD of 110 nm, leading to a drastically reduced tensile strength of only 3 MPa in this specific case.

All displayed films show a more or less mesoporous structure which ranges from quasi-dense to dust-like with a smooth transition. However, with regard to their suitability as an adhesive layer, the structures can be roughly divided into three categories:

- Quasi-dense films are characterized by a combination of a low MAD, typically below 30 nm, and a FSA of less than 20%. Figure 7a shows a film in the transition region between quasi-dense and mesoporous morphologies. Due to the extremely narrow capillaries between the agglomerates, the structure exhibits a minimal permeability for the polymer melt. Figure 7b illustrates the characteristic fracture pattern of a quasi-dense film. The polymer melt did not infiltrate the porous structure. The image was captured after mechanical testing and reveals that if infiltration occurred at all, it was limited to isolated regions with slightly wider capillaries. On the polymer side of the fracture surface, shown in Figure 7c, it is apparent that the polymer melt spreads only across the surface without penetrating the pore network through capillary action. The interface failed adhesively at the SiO_2_–polymer boundary, resulting in negligible interfacial strength. The deposition of similar quasi-dense films using microwave plasma-enhanced chemical vapor deposition (PECVD) was first reported by Dreher et al. in 2009 [47]. Subsequent studies by Laux et al. [23] confirmed that such films are not well suited as adhesion-promoting interlayers, particularly in polymer–metal hybrid systems fabricated by injection molding, presumably due to their low porosity and inter-columnar spacing.
- Mesoporous adhesion films exhibit intermediate morphological characteristics with MAD values typically ranging from 30 to 90 nm and the FSA between 25% and 45%. An example of such a mesoporous film is shown in Figure 7d. These structures possess an open-pore network that allows infiltration of the polymer melt during overmolding, which could be also shown in previous studies [16,22,23]. As a result, mixed-mode fracture patterns are commonly observed as can be seen in Figure 7e,f. Depending on local variations in pore size and film thickness, both adhesive and cohesive failure mechanisms can be identified. Residual SiO_2_ material can be found on one or both sides of the fracture surface, indicating good interlocking at the interface which results in high interfacial adhesion strengths up to 30 MPa for an SiO_2_–PPS interface.
- Dust-like films, by contrast, typically exhibit MAD greater than 90 nm in combination with a high FSA, as exemplified in Figure 7g. These structures tend to form as flaky or loosely packed dust-like films, and this characteristic becomes more pronounced as both FSA and MAD increase. Due to the weak interconnection between individual agglomerates, these films are referred to as dust-like in the context of this study.
  When such films are overmolded during injection molding, the polymer melt infiltrates the porous structure. However, due to the low mechanical cohesion within the SiO_2_ structure, mainly caused by the weak interconnection between individual agglomerates, failure occurs cohesively within the film itself. This behavior is clearly visible in the fracture surfaces shown in Figure 7h,i, where significant residues of SiO_2_ agglomerates are present on both sides of the fracture interface.

In addition to the layer categories defined in the previous section, the trend shows that the FSA tends to increases with an increasing agglomerate diameter. The correlation between FSA and MAD roughly follows the trend line:

(7) $$\mathrm{FSA}\approx \frac{1}{4}\mathrm{MAD}+15$$

where the upper limit of a achievable FSA for a given MAD is limited by

(8) $${\mathrm{FSA}}_{max}\approx \frac{1}{4}\mathrm{MAD}+30$$

and the minimum achievable FSA is limited by

(9) $${\mathrm{FSA}}_{min}\approx \frac{1}{4}\mathrm{MAD}$$

Combinations above and below this threshold were not observed.

### 3.1. Results of the Regression Analysis

Fitting the models using MLR in combination with an augmented backward elimination approach, as described in Section 2.2, good regression models could be derived for all three responses: FSA, MAD, and *h*. Table 3 lists the coefficients along with their corresponding *p*-values. To check if the residuals are normally distributed, the deleted studentized residuals were ordered by size for each response and plotted against their normal probability. Considering seven input variables in each regression model complicates the presentation of the results. The regression models for the responses FSA, MAD, and *h* are presented graphically using effect plots. The plots display the respective response and the 95 percent confidence interval for each input variable, with all other variables fixed at their average values. For each input variable, the model was checked for fundamental changes in its trend within the investigated parameter space. By determining the largest gradient, parameter settings could be identified where the model is most sensitive to changes in each influencing variable. This involved deriving the model equation according to the main effect and performing an extreme value analysis to identify points of maximum or minimum rate of change. The responses at these points of maximum and minimum gradient are plotted in the upper part of the diagram. All input parameters that do not influence the slope gradient were set to their center point to improve comparability, as shown in Table 1. Responses that change algebraic signs within the analyzed parameter space are marked with an asterisk symbol (*). For these parameters, an extended analysis of the response is necessary to avoid drawing conclusions that are only valid in a specific model range.

#### 3.1.1. Free Surface Area

**Figure 8:**
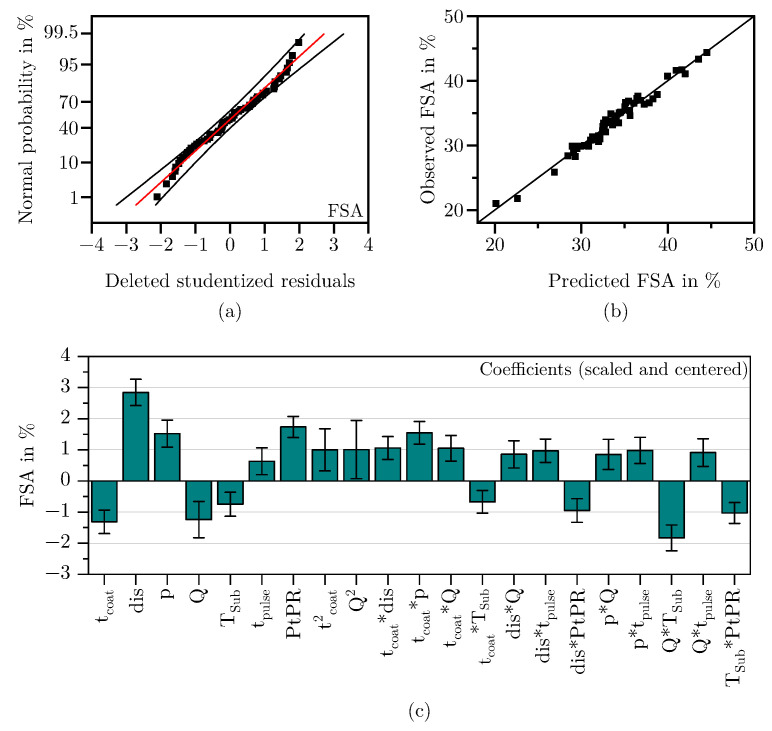
Residuals normal probability plot (**a**), observed vs. predicted scatter plot (**b**), and scaled and centered coefficients (**c**) for the response FSA.

The response FSA required the highest number of coefficients to achieve good model quality. After backward elimination, the regression equation comprises seven main effects, two quadratic effects and 12 interactions as can be seen in Figure 8c. Six of the seven main effects were highly significant with *p*-values < 0.001.

Figure 8a shows the deleted studentized residuals, ordered by size and plotted against their normal probability. Since they follow a straight line and no outliers exceed the ±4 SD mark, it can be assumed that the residuals are approximately normally distributed [48]. Additionally, Figure 8b demonstrates that the observed and predicted values of the model align well. With a Q^2^ of 0.91, an adjusted R^2^ of 0.96, and a model validity of 0.89 (see Table 3), it can be concluded that the selected predictors describe the response FSA well and that the model is of high quality [35].

**Figure 9:**
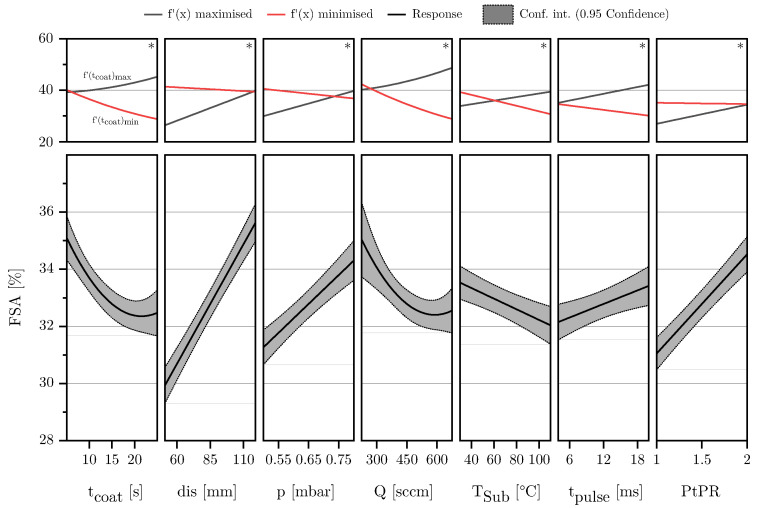
The lower part of the diagram shows a standard effect plot for the response FSA, illustrating the influence of the respective factor while all other variables are held at their center point. The upper part of the diagram presents the results of an extreme value analysis, displaying the effect of the respective factor when all other parameters are set to maximize or minimize its influence on the output variable. This corresponds to the maximum and minimum slope of the effect curve. If the sign of the slope changes within this range analysis, an asterisk symbol (*) is added in the upper right corner of the diagram, indicating a fundamental change in the factor’s effect.

Figure 9 shows the response of the FSA as a function of the individual input parameters. Upon deriving the model equation according to the respective input variables, it is evident that the response FSA exhibits sign changes across nearly all input variables within the model space. This necessitates a thorough and precise analysis of the results.

Within the analyzed model space, the distance exhibits the greatest effect on the FSA, highly interacting with the parameters $t_{\mathrm{coat}}$, *Q*, $t_{\mathrm{pulse}}$, and PtPR. This influence peaks at a combination of long coating times, high flow rates, long pulse durations, and low PtPRs. In contrast, it diminishes with opposite settings, resulting in a slight decrease in FSA with increasing coating distance.

The parameter pause-to-pulse ratio similarly exerts a significant influence, with higher PtPRs leading to a notable increase in FSA. Counteracting interactions with the parameters dis and $T_{\mathrm{Sub}}$ are observed, maximizing influence at minimum settings of these parameters. Interestingly, the influence of the PtPR is minimal at maximum distance and flow rate.

Examining the coating time factor reveals an initial significant decrease in FSA, which then levels off into a plateau where the FSA does not decrease any further. The influence of coating time on FSA is contingent upon distance, pulse duration, and flow rate. At high distances combined with high chamber pressures and flow rates, coating time exerts the strongest effect on FSA, initially causing a significant decrease before stabilizing on a plateau. Conversely, minimizing the parameters *dis*, $t_{\mathrm{pulse}}$, and *Q* reverses this effect, resulting in a slight increase in FSA with increased coating time.

**Figure 10:**
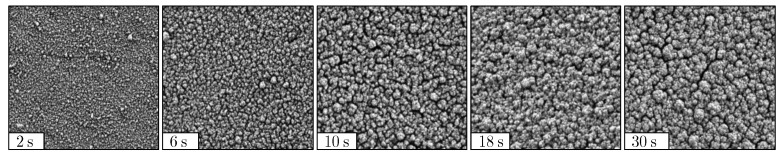
Different film structures at constant deposition parameters for a increased coating time $t_{\mathrm{coat}}$, with dis=85 mm, p=0.55 mbar, Q=450 SCCM, $T_{\mathrm{Sub}}=27\;{}^{\circ}C$, $t_{\mathrm{pulse}}=10$ ms, PtPR=1.

Figure 10 presents SEM images of a time series conducted under otherwise identical coating parameters. The analyzed series is based on the center point of the DoE, which was extended by additional data points for this time series.

Image analysis reveals that the FSA initially exhibits high values and that the structure becomes increasingly compact at longer coating times. After approximately 18 s, compacting stagnates, and the FSA no longer decreases significantly. This observation aligns with the predictions from the effect plot in Figure 9.

Furthermore, it is evident that the film structure initially consists of small agglomerates, which merge into larger structures over time. This process correlates with the decrease in FSA: At the beginning, films with a high FSA are deposited, consisting of fine agglomerates. As deposition progresses, the growth of these agglomerates leads to structural densification and, consequently, a reduction in FSA.

This growth behavior is noticed for most parameter combinations. However, an exception is observed for parameter combinations involving a small coating distance, short pulse time, and low flow rate. In these cases, predominantly dense layers are deposited, as described earlier. It is hypothesized that as the film thickness increases, this dense structure begins to break up, which is reflected in a rising FSA. Further research is needed to confirm this assumption.

The chamber pressure has a medium influence on the FSA. An increase in *p* generally leads to a higher FSA, an effect that is particularly amplified with an increasing of *Q*, $t_{\mathrm{pulse}}$, and $t_{\mathrm{coat}}$. This aligns with observations regarding the MAD, suggesting that increased gas-phase reactions at higher pressures result in less particle orientation and condensation on the substrate, which leads to the deposition of more porous morphologies, and thus a higher FSA (see also Section 3.1.2).

The influence of the flow rate is dominated by relatively strong interactions with the parameters $t_{\mathrm{coat}}$, dis, *p*, and $T_{\mathrm{Sub}}$, as well as a quadratic effect. Considering the center point of the model, the FSA response is strongest at low to medium flow rates, with low flow rates leading to layers with higher porosity. Increasing flow rates initially cause a significant decrease in FSA. Once the flow rate exceeds 500 SCCM, no further decrease is observed, and the FSA reaches a plateau where the effect is no longer significant, and the changes up to the upper model boundary lie within the confidence interval. At low coating times, distances, and pressures combined with high substrate temperatures, the influence is quantitatively strongest, but the basic behavior remains as described above. However, when considering high coating times, distances, and pressures combined with low substrate temperatures, the effect of the flow rate fundamentally reverses. In this parameter combination, the FSA increases with increasing flow rate.

The parameters substrate temperature and pulse duration exert the least influence on the FSA. Generally, FSA decreases with increasing $T_{\mathrm{Sub}}$, particularly pronounced at high flow rates, while it slightly increases with increasing pulse duration, especially at high distances, pressures, and flow rates.

#### 3.1.2. Mean Agglomerate Diameter

**Figure 11:**
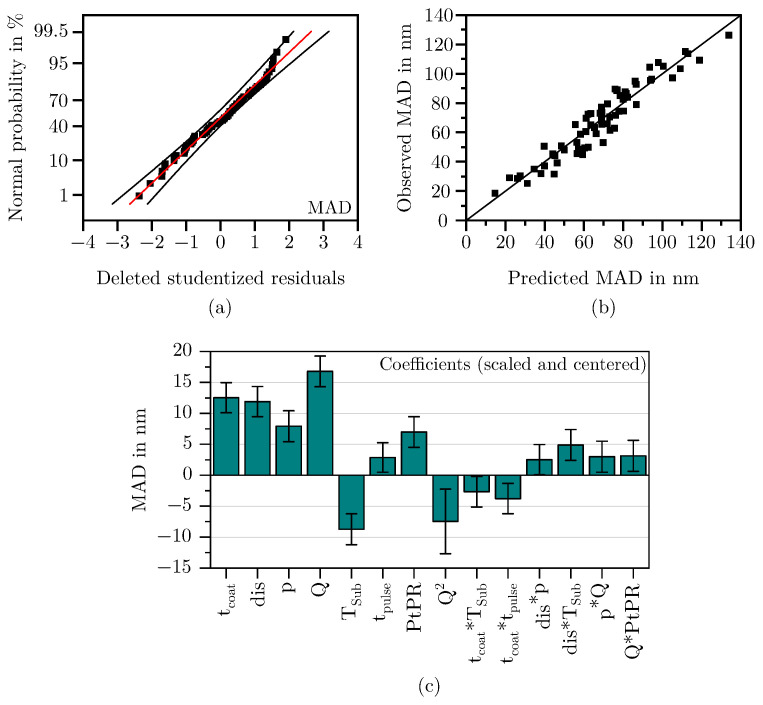
Residuals normal probability (**a**), observed vs. predicted scatter plot (**b**), and scaled and centered coefficients (**c**) for the response MAD.

The model for the response MAD could be reduced to a total of seven main effects, one quadratic effect and six interactions. As can be seen in Figure 11a, the residuals are normally distributed and the values predicted by the model align well with the values observed during the DoE (see Figure 11b). The scaled and centered coefficients are shown in Figure 11c. The coefficients of the model equation and the corresponding *p*-values can be found in Table 3. The model is of high quality, with a Q^2^ of 0.85, an R^2^ adjust of 0.88, and a model validity of 0.63, so that also for the response MAD it can be concluded that the selected predictors describe the response well.

**Figure 12:**
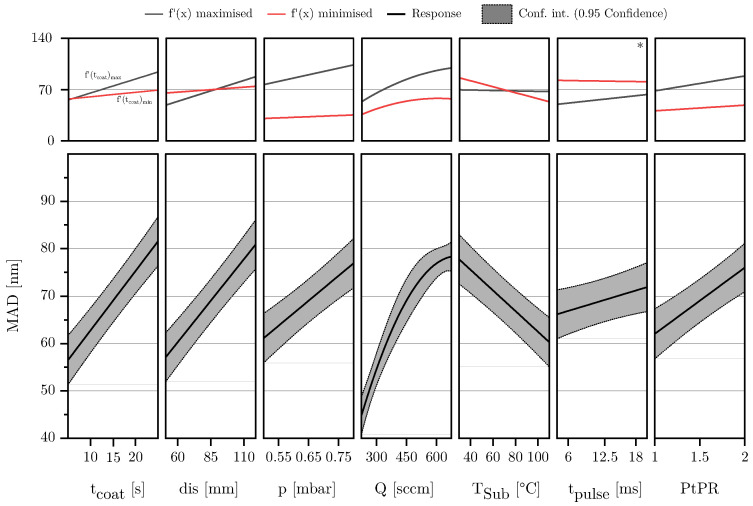
The lower part of the diagram shows a standard effect plot for the response MAD, illustrating the influence of the respective factor while all other variables are held at their center point. The upper part of the diagram presents the results of an extreme value analysis, displaying the effect of the respective factor when all other parameters are set to maximize or minimize its influence on the output variable. This corresponds to the maximum and minimum slope of the effect curve. If the sign of the slope changes within this range analysis, an asterisk symbol (*) is added in the upper right corner of the diagram, indicating a fundamental change in the factor’s effect.

As shown in Figure 12 the MAD is mostly affected by the flow rate, followed by the parameters $t_{\mathrm{coat}}$ and dis. The strong effect of *Q* increases even further at high pressures and PtPRs. However, due to a pronounced quadratic effect, the agglomerate growth is significantly reduced at very high flow rates. This suggests that the plasma power of the coating array becomes insufficient at these levels to fully polymerize the precursor HMDSO.

The parameters coating time and chamber pressure exerts the second strongest influence on the MAD. Both parameters show a similarly strong impact, especially at the center point of the model. When evaluating the influence of coating time on the MAD together with its influence on the FSA, it becomes evident that for more or less all parameter combinations, initially, the deposited film structures are composed of small agglomerates, which then merge into increasingly larger structures over time. As described in Section 3.1.1, this is accompanied by a significant initial decrease in the FSA, indicating a strong consolidation of the film structure in the early stages of the deposition. For the FSA, a plateau is observed beyond a certain point, where no further decrease occurs. Interestingly, the MAD shows no such plateau; it increases continuously with coating time within the investigated parameter space. The corresponding extreme value analysis further confirms that this increase occurs across all tested parameter combinations, with the effect never reversing.

The agglomerate enlarging effect of the chamber pressure can be explained as an increase in pressure increases particles per volume in the gas space, which leads to the formation of large agglomerates as the probability of particle collision and, thus, caking increases. In combination with low substrate temperatures, the agglomerates formed in this way can no longer subsequently restructure on the surface, as the surface mobility is restricted. Increasing the distance further intensifies this effect. Particles have more time in the gas space to bake together and form large agglomerates before hitting the substrate surface. Consequently, the highest average agglomerate diameters of up to 130 nm were observed with long coating times, considerable substrate distances, high chamber pressures, medium to high flow rates, and relatively low substrate temperatures. However, SEM images also showed that agglomerates larger than approximately 90 nanometers tend to be deposited in the form of dust. This assumption based on the optical analysis has already been confirmed by Laux et al. [23] on the basis of adhesive strength tests.

**Figure 13:**
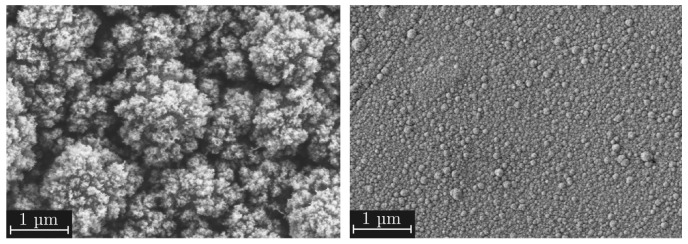
(**left**) Film morphology with a high MAD of 126 nm ($t_{\mathrm{coat}}=25$ s, dis=119 mm, p=0.8 mbar, Q=675 SCCM, $T_{\mathrm{Sub}}=30\;{}^{\circ}C$, $t_{\mathrm{pulse}}=4$ ms, PtPR=2); (**right**) Film morphology with a low MAD of 44 nm ($t_{\mathrm{coat}}=25$ s, dis=51 mm, p=0.5 mbar, Q=675 SCCM, $T_{\mathrm{Sub}}=110\;{}^{\circ}C$, $t_{\mathrm{pulse}}=4$ ms, PtPR=1).

On the other hand, high substrate temperatures lead to the deposition of a more coherent and dense morphology, favoring the formation of small and compact agglomerates as can be seen in the right image of Figure 13. As high surface temperatures enhance adatom mobility, the individual reactive particles bond more easily, compacting the deposited film/agglomerates and reducing the volume accordingly. The observations made with regard to the influence of the substrate temperature coincide with the well-known structure zone model [29] and are also observed for porous PECVD silica films in the investigations of Borer et al. [49]. Long coating times and small substrate distances further amplify this effect.

The parameters pulse duration and pause-to-pulse-ratio have the slightest impact on the MAD. In the case of the pulse duration, the effect can just barely be regarded as significant. For coating durations longer than 20 s, the effect is almost zero.

#### 3.1.3. Film Thickness

**Figure 14:**
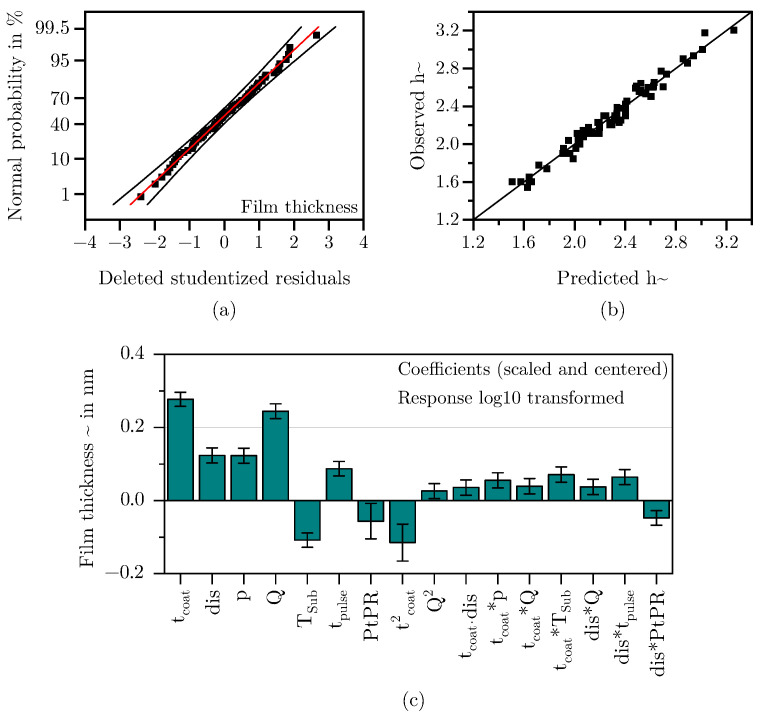
Residuals normal probability (top left), observed vs. predicted regression scatter plot (top right) and scaled and centered coefficients for the response *h*.

In the case of the film thickness *h*, a 10 Log(Y) transformation was performed to obtain a normally distributed data set. The model for the response *h* could be reduced to a total of six main effects, two quadratic effect and eight interactions. As can be seen the residuals are normally distributed (see Figure 14a) and the values predicted by the model align well with the values observed during the DoE (see Figure 14b). The scaled and centered coefficients are shown in Figure 14c. The coefficients of the model equation and the corresponding *p*-values can be found in Table 3. The model is of high quality, with a Q^2^ of 0.85, an R^2^ adjust of 0.88 and a model validity of 0.62. The slightly lower model validity can be explained by the high sensitivity of the statistical tests used. Additionally, not every parameter setting was tested with multiple replicates, although the existing replicates demonstrate very good reproducibility [50]. Overall it can be concluded that the selected predictors describe the response well.

**Figure 15:**
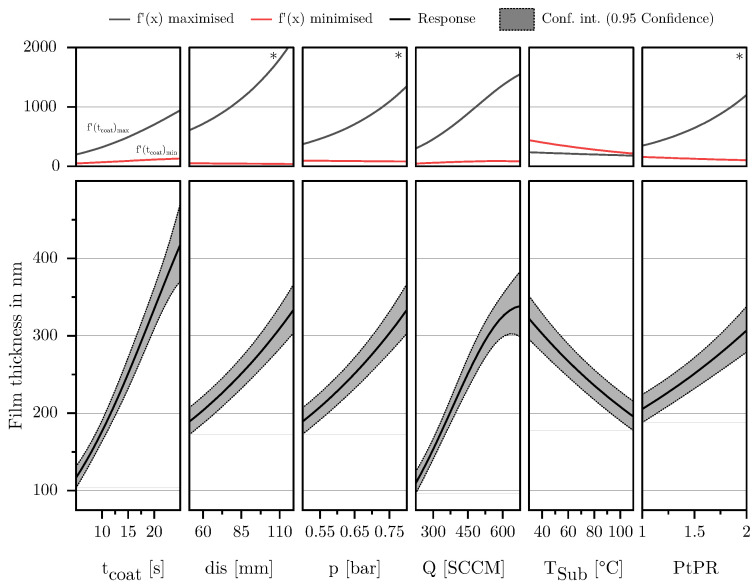
The lower part of the diagram shows a standard effect plot for the response *h*, illustrating the influence of the respective factor while all other variables are held at their center point. The upper part of the diagram presents the results of an extreme value analysis, displaying the effect of the respective factor when all other parameters are set to maximize or minimize its influence on the output variable. This corresponds to the maximum and minimum slope of the effect curve. If the sign of the slope changes within this range analysis, an asterisk symbol (*) is added in the upper right corner of the diagram, indicating a fundamental change in the factor’s effect.

As shown in Figure 15 the film thickness *h* is mostly influenced by the two factors $t_{\mathrm{coat}}$ and *Q*. Considering the effect of the coating time, it can be seen that *h* increases over time regardless of the settings of the other parameters. The gradient of the curve corresponds to the deposition rate in nanometers per second. In addition it can be seen that, within the investigated parameter space, the deposition rate varies significantly. Highest deposition rates are achieved with combinations of a high *Q* and a large $t_{\mathrm{coat}}$.

As the flow rate defines the amount of precursor introduced per unit time, it has a significant influence on the deposition rate and, consequently, the film thickness. Upon closer examination, it is evident that *h* does not continue to increase as expected at high flow rates, which is indicated by a strongly negative quadratic interaction in the regression model. This effect was also observed for the FSA and the MAD. Together, these observations strongly suggest that the phenomenon is due to a lack of plasma energy in the system, leading to an inadequate conversion of the precursor HMDSO, which has already been shown by Dreher et al. [47] in studies on the deposition of dense SiO_2_ films in microwave-induced PECVD.

The parameters distance and pressure have a moderate influence on the film thickness. With increasing distance and pressure, *h* generally increases at most points within the parameter space. However, the influence of distance becomes very strong at high pressures, flow rates, and pause-to-pulse ratios, surpassing even the effect of coating time in this marginal area of the model. This is because these parameter combinations favor the deposition of powdery films with large and loosely dense agglomerates. The volume of the deposited layers increases exponentially as can be seen in the left SEM picture of Figure 13. In contrast, for parameter combinations of low pressures, flow rates, and pause-to-pulse ratios, the coating distance has a very weak and simultaneously negative influence on the film thickness. Due to the low pressures and flows, there is a low collision rate. Due to the low pause-to-pulse ratio, small agglomerates tend to form in the gas phase, which can be well sorted and baked together at the substrate surface due to the prevailing high plasma power per precursor flow.

Similar considerations apply to the parameter chamber pressure. In most parameter combinations, pressure has a positive influence on the film thickness, comparable to the effect of distance at the center point of the model. The coating process is most sensitive to pressure in combinations of a high *Q* and a high PtPR. Here, too, the sign is reversed to minimize interactions with Q and pause-to-pulse ratios. The underlying phenomena are analogous to the explanation in the previous paragraph.

At low pause-to-pulse ratios, the plasma work introduced is maximal. Consequently, this favors all phenomena of plasma polymerization and layer deposition, resulting in layers deposited at low pause-to-pulse ratios tending to be denser and exhibiting less volumetric layer buildup over time. At high pause-to-pulse ratios, the average plasma work and the agglomerates tend to be less dense in the gas phase. This effect is most substantial at high pressures and low substrate temperatures, as high pressures are synonymous with many particles/volume elements. Lack of plasma power here results in poor conversion of process gases. Additionally, if substrate temperatures are low, the agglomerates formed in the gas phase cannot re-compact on the surface and tend to deposit powder.

As the only parameter, $T_{\mathrm{Sub}}$ has a negative influence on the film thickness in all parameter combinations. The effect is maximized in combination with a high PtPR. With increasing $T_{\mathrm{Sub}}$, the deposition rate decreases significantly. The observed phenomenon aligns with fundamental findings from studies investigating the effect of substrate temperature on the deposition rate in plasma polymerization [51]. It is assumed that this is due to the higher mobility of initially loosely bound adatoms to the surface, which allows them to diffuse better and regroup on the substrate surface, favoring the formation of denser layers and thereby reducing the deposition rate. More specific regarding the deposition of SiO_2_ Borer et al. [49] observe a similar phenomenon, investigating the influence of substrate temperature on morphology of SiO_x_ films deposited on particles by PECVD.

The factor $t_{\mathrm{pulse}}$ showed no significant influence in its main effect or interactions and was therefore removed from the regression model of the MAD.

### 3.2. Description of Characteristic Model Areas Using 4D Contour Plots

**Figure 16:**
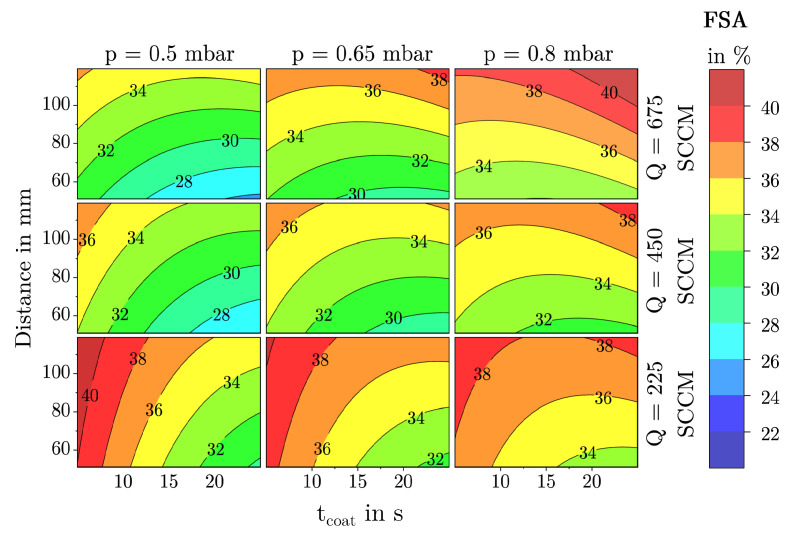
4D contour plot of the response FSA as a function of the factors $t_{\mathrm{coat}}$, dis, *Q*, and *p* with all other factors held constant at their center point.

**Figure 17:**
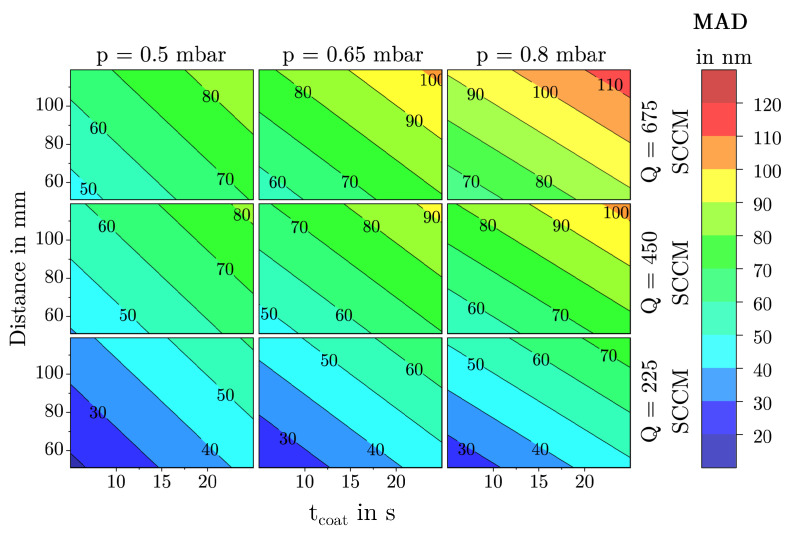
4D contour plot of the response MAD as a function of the factors $t_{\mathrm{coat}}$, dis, *Q*, and *p* with all other factors held constant at their center point.

**Figure 18:**
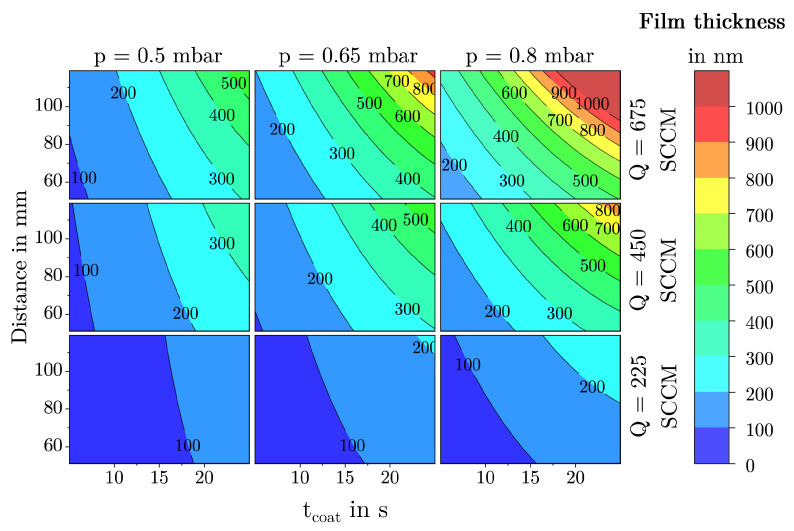
4D contour plot of the response ***h*** as a function of the factors $t_{\mathrm{coat}}$, dis, *Q*, and *p* with all other factors held constant at their center point.

To make fundamental film growth phenomena easier to explain, the following discussion focuses on the four most influential factors $t_{\mathrm{coat}}$, *p*, dis, and *Q*. Using 4D contour plots as shown in Figure 16, Figure 17 and Figure 18, significant structural regions within the deposition model can be visualized. For the creation of these plots, the factors $T_{\mathrm{Sub}}$, $t_{\mathrm{pulse}}$, and PtPR were set to their respective center points as shown in Table 1. The color scale ranges from blue (low values) through green to red (high values). Since all three diagrams use identical axis scaling for the input parameters, a direct comparison of the deposition areas is possible.

The observed film structures range from quasi-dense films to dust-like formations. This transition is gradual and cannot be precisely quantified. A comparison of the 4D contour plots for the MAD and *h*, as shown in Figure 17 and Figure 18, reveals a clear correlation between these two responses. Low MAD values are often associated with a low deposition rate or film thickness, whereas large, dust-like agglomerates lead to a high deposition rate and, consequently, thicker films. Interestingly, this correlation does not apply to the response FSA, as the model shows that films with a specific FSA can consist of either small or large agglomerates as can be seen in Figure 16.

**Figure 19:**
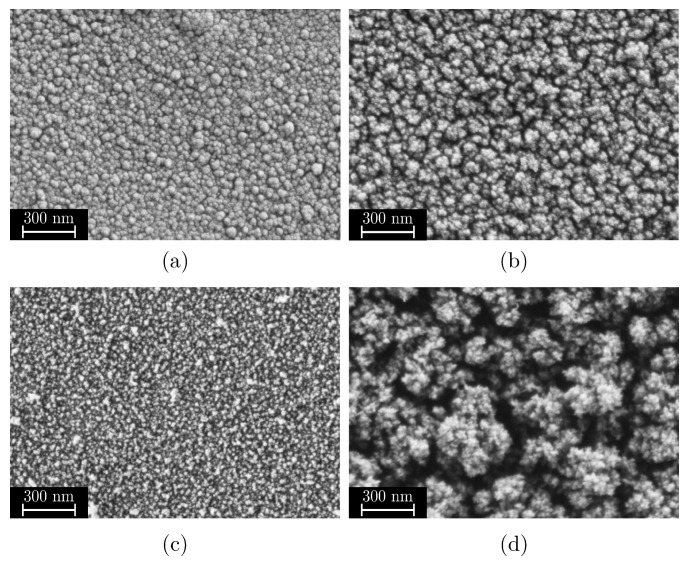
Change in film structure depending on the parameters $t_{\mathrm{coat}}$, dis, *p* and *Q*. (**a**) Quasi-dense structures with $t_{\mathrm{coat}}=25$ s, dis=51 mm, p=0.5 mbar, Q=225 SCCM. (**b**) Regular structures as reported in [16,22] with $t_{\mathrm{coat}}=15$ s, dis=85 mm, p=0.65 mbar, Q=450 SCCM. (**c**) High FSA composed of small agglomerates with $t_{\mathrm{coat}}=5$ s, dis=119 mm, p=0.8 mbar, Q=225 SCCM. (**d**) High FSA composed of dust-like structures with $t_{\mathrm{coat}}=25$ s, dis=119 mm, p=0.8 mbar, Q=675 SCCM.

Quasi-dense films with a low FSA, as shown in Figure 19a, predominantly form in the blue regions of the diagrams. These structures are characterized by low to medium agglomerate diameters and low FSA. Such closed films are mainly formed at low pressure, short distances, low flow rates, and medium to high coating times (in this investigation above 15 s). Due to their dense structure, these films are difficult to infiltrate for polymer melts and in addition offer little surface area for the formation of a form closure which is necessary for the adhesion effect of the films investigated and therefore are unsuitable as adhesion films for thermoplastic injection molding as can be seen in Figure 6 and could also be shown in previous investigations [23].

Films with a high FSA are found in the red regions of the FSA contour plot. They can consist of both small to medium or large agglomerates. Structures with high FSA and small agglomerates, as shown in example in Figure 19c, form at low pressure, low flow rates, large coating distances, and short coating times. The large coating distance promotes gas-phase reactions, while the short coating time prevents further compacting of particles on the substrate surface. Interestingly, the model demonstrates that these films gradually transform into denser structures with prolonged deposition time. This occurs as the agglomerates continue to grow, cluster, and compact over time.

On the other hand, films with a high FSA can also form at high pressures and flow rates in combination with a large coating distance as shown in Figure 19d. In this case, a high FSA is associated with large MAD values and high deposition rates hence film thicknesses. As known from previous studies, such films exhibit a dust-like structure and are unsuitable as adhesion films.

Mesoporous films with a medium FSA of around 40 to 90 nanometers represent a transition area between the previously described extremes and were the focus of this study. These structures, corresponding to those described by Emmerich et al. [16] are located in the green to yellow regions of the analysis diagrams. Previous investigations by Laux et al. [22,23] demonstrated that for a PPS–SiO_2_–aluminum interface with glass fiber-reinforced PPS, interfacial shear strengths of up to 28 MPa can be achieved. Optimal adhesion was observed at an FSA between 33 and 38 percent and a mean agglomerate size of 60 to 90 nm. Both lower and higher FSAs or agglomerate sizes resulted in significantly reduced adhesive strengths. These results indicate that both FSA and agglomerate morphology play a key role in interfacial performance. It remains to be shown whether these trends are transferable to other polymer systems and whether the deposition model developed here can be used to systematically tailor interfacial layers for specific material combinations.

## 4. Discussion

This study examined the growth behavior of mesoporous SiO_2_ films deposited by microwave PECVD using a Design of Experiments approach. The deposited films were characterized based on their structural properties, such as free surface area, mean agglomerate diameter, and film thickness. All parameters were quantitatively analyzed using digital image segmentation and white light interferometry. Additionally, all films were subsequently overmolded via injection molding and mechanically tested, enabling a direct correlation between their morphological characteristics and performance as adhesion-promoting interlayers. The mesoporous silica films exhibited a wide range of morphologies. However, not all structures within the investigated parameter space fulfilled the criteria for mesoporosity as defined by Emmerich et al. [16]. At the edges of the parameter space, quasi-dense and dust-like film growth was observed. Mechanical characterization showed that these types of structures provide limited adhesion performance. To achieve a good model fit, all seven influencing factors had to be considered for the responses FSA and MAD. Only the pulse duration showed no significant effect on film thickness. The analysis revealed strong interactions among factors, meaning that the effect of individual parameters often depended heavily on the settings of others. Although the complexity made general statements difficult and required a holistic interpretation of the regression models, several general trends consistent across most parameter combinations were identified. Among all parameters, the coating time and coating distance exhibited the most consistent and significant effects. Short coating times typically resulted in films composed of small agglomerates with high surface area, although the limited deposition duration led to thin films. With increasing coating time, the agglomerates grew larger, the film structure became denser, and the FSA decreased. Smaller distances reduced gas-phase reactions and promoted surface reactions, generally resulting in films with lower FSA and MAD and reduced deposition rates. In contrast, larger distances encouraged gas-phase reactions, often leading to the formation of larger agglomerates and higher FSA. The impact of coating distance on film thickness depended strongly on other parameters such as pressure, flow rate, and the pause-to-pulse ratio. The precursor flow rate primarily affected MAD and film thickness, while the chamber pressure played a critical role in determining FSA and deposition rates. Lower pressures led to denser films with reduced growth rates, while higher pressures increased deposition but tended to produce dust-like morphologies. The pause-to-pulse ratio significantly influenced FSA, and substrate temperature had a strong effect on both MAD and thickness. The influence of other parameters varied and is discussed in detail in Section 3.1. The application of digital image segmentation proved effective for film characterization. The derived regression models showed good accuracy, indicating that the selected process parameters were appropriate. Due to the complexity of factor interactions, the DoE approach was well suited to describe the non-linear effects governing mesoporous film formation. These findings offer a robust foundation for further investigations into the adhesion behavior of mesoporous SiO_2_ films. Observing quasi-dense and dust-like morphologies at the edges of the parameter space confirms that the explored parameter range covers most of the conditions under which mesoporous film growth can occur. Compared to previous work by Emmerich et al. [16], the range of film structures was significantly extended. Previously unreported adhesion-promoting layers were identified, which appear highly promising for future investigation. This study makes a substantial contribution to understanding mesoporous SiO_2_ film deposition by microwave PECVD. It provides a comprehensive basis for future research on the suitability of such films as interfacial adhesion films in injection molding or similar applications.

## Figures and Tables

**Table 1:** Deposition parameter levels used for the DoE.

Parameter	Abbreviation	Unit	Low	Mid	High
Coating time	$t_{\mathrm{coat}}$	s	5	15	25
Distance	*dis*	mm	51	85	119
Chamber pressure	*p*	mbar	0.5	0.65	0.8
Flow rate	*Q*	SCCM	225	450	675
Substrate temperature	$T_{\mathrm{Sub}}$	°C	30	70	110
Pulse duration	$t_{\mathrm{pulse}}$	ms	4	12	20
Pause-to-pulse ratio	*PtPR*		1	1.5	2
**Response**					
Free surface area	FSA	percentage			
Mean agglomerate diameter	MAD	nm			
Film thickness	*h*	nm			

**Table 2:** Injection molding parameters for the production of the hybrid PPS–aluminum tensile bars.

Parameter	Value	Unit
Melt temperature	340	°C
Mold temperature	155	°C
Dosing volume	25	cm^3^
Injection rate	60	cm^3^/s
Switching point	12	cm^3^
Holding pressure	430	bar
Holding pressure time	20	s
Total cooling time	40	s

**Table 3:** Model coefficients and dedicated *p*-values.

	Film Thickness			FSA			MAD		
Variable	Coeff. SC	*p*-Value		Coeff. SC	*p*-Value		Coeff. SC	*p*-Value	
Constant	2.39996			32.7858			69.0311		
$t_{\mathrm{coat}}$	0.277175	2.12 × $10^{-36}$	***	−1.3126	2.26 × $10^{-8}$	***	12.527	3.51 × $10^{-14}$	***
dis	0.123398	2.13 × $10^{-17}$	***	2.84606	4.71 × $10^{-16}$	***	11.904	1.82 × $10^{-13}$	***
*p*	0.122994	2.15 × $10^{-17}$	***	1.52006	2.01 × $10^{-8}$	***	7.9097	5.90 × $10^{-8}$	***
*Q*	0.24465	3.99 × $10^{-32}$	***	−1.24068	1.19 × $10^{-4}$	***	16.7836	1.06 × $10^{-18}$	***
$T_{Sub}$	−0.108177	6.39 × $10^{-16}$	***	−0.746949	3.70 × $10^{-4}$	***	−8.72345	4.25 × $10^{-9}$	***
$t_{\mathrm{pulse}}$			-	0.633215	5.35 × $10^{-3}$	**	2.86518	1.93 × $10^{-2}$	*
PtPR	0.087169	2.14 × $10^{-12}$	***	1.73624	1.47 × $10^{-12}$	***	6.98881	6.00 × $10^{-7}$	***
$t_{\mathrm{coat}}^{2}$	−0.0562705	2.39 × $10^{-2}$	*	0.999879	4.87 × $10^{-3}$	**			-
${\mathrm{dis}}^{2}$			-			-			-
$p^{2}$			-			-			-
$Q^{2}$	−0.115093	2.65 × $10^{-5}$	***	1.00844	3.56 × $10^{-2}$	*	−7.4682	5.82 × $10^{-3}$	**
$T_{\mathrm{Sub}}^{2}$			-			-			-
$t_{\mathrm{pulse}}^{2}$			-			-			-
${\mathrm{PtPR}}^{2}$			-			-			-
$t_{\mathrm{coat}}\cdot \mathrm{dis}$	0.025953	1.48 × $10^{-2}$	*	1.05831	1.04 × $10^{-6}$	***			-
$t_{\mathrm{coat}}\cdot p$			-	1.54715	1.87 × $10^{-10}$	***			-
$t_{\mathrm{coat}}\cdot Q$	0.035658	1.15 × $10^{-3}$	**	1.04961	8.32 × $10^{-6}$	***			-
$t_{\mathrm{coat}}\cdot T_{\mathrm{Sub}}$			-	−0.671548	6.04 × $10^{-4}$	***	−2.67023	3.51 × $10^{-2}$	*
$t_{\mathrm{coat}}\cdot t_{\mathrm{pulse}}$			-			-	−3.78306	3.26 × $10^{-3}$	**
$t_{\mathrm{coat}}\cdot \mathrm{PtPR}$			-			-			-
dis·p	0.055434	1.67 × $10^{-6}$	***			-	2.51179	4.42 × $10^{-2}$	*
dis·Q	0.039203	4.28 × $10^{-4}$	***	0.855687	3.17 × $10^{-4}$	***			-
$\mathrm{dis}\cdot T_{\mathrm{Sub}}$			-			-	4.87852	2.63 × $10^{-4}$	***
$\mathrm{dis}\cdot t_{\mathrm{pulse}}$			-	0.971383	6.37 × $10^{-6}$	***			-
dis·PtPR	0.071159	6.51 × $10^{-9}$	***	−0.949231	1.27 × $10^{-5}$	***			-
p·Q	0.037407	7.48 × $10^{-4}$	***	0.853839	1.01 × $10^{-3}$	**	2.99637	2.06 × $10^{-2}$	*
$p\cdot T_{\mathrm{Sub}}$			-			-			-
$p\cdot t_{\mathrm{pulse}}$			-	0.982965	3.24 × $10^{-5}$	***			-
p·PtPR	0.0640944	6.40 × $10^{-8}$	***			-			-
$Q\cdot T_{\mathrm{Sub}}$			-	−1.83086	9.06 × $10^{-11}$	***			-
$Q\cdot t_{\mathrm{pulse}}$			-	0.913311	1.86 × $10^{-4}$	***			-
Q·PtPR			-			-	3.13988	1.55 × $10^{-2}$	*
$T_{\mathrm{Sub}}\cdot t_{\mathrm{pulse}}$			-			-			-
$T_{\mathrm{Sub}}\cdot \mathrm{PtPR}$	−0.0476075	1.32 × $10^{-5}$	***	−1.02951	3.22 × $10^{-7}$	***			-
$t_{\mathrm{pulse}}\cdot \mathrm{PtPR}$			-			-			-
Q2	0.95			0.91			0.85		
R2	0.97			0.97			0.91		
R2 adj.	0.96			0.96			0.88		
Conf.	0.95			0.95			0.95		

*** *p* < 0.001; ** *p* < 0.01; * *p* < 0.05; - non significant *p* > 0.05

## Data Availability

The original contributions presented in this study are included in the article. Further inquiries can be directed to the corresponding authors.

## Abbreviations

The following abbreviations are used in this manuscript:

AFM	Atomic force microscopy
CCF	Face-centered central composite design
dis	here: Distance between precursor feed and substrate
FSA	Free surface area
*h*	Film thickness
HMDSO	Hexamethyldisiloxane
ICT	Fraunhofer Institute for Chemical Technology
MAD	Mean agglomerate diameter
MLR	Multiple linear regression
*p*	here: Array pressure
PECVD	Plasma-enhanced chemical vapor deposition
MPECVD	Microwave plasma-enhanced chemical vapor deposition
PID	Proportional–integral–derivative
PtPR	Pause-to-pulse-ratio
*Q*	Flow rate
Q^2^	Predictive relevance
*R*	Dynamic range of standard deviation
R^2^	Coefficient of Determination
SCCM	Standard cubic centimeters per minute
SED	Secondary electron detector
SEM	Scanning electron microscopy
SLPM	Standard liter per minute
$t_{\mathrm{coat}}$	Coating time
$t_{\mathrm{pulse}}$	Pulse time
$T_{\mathrm{Sub}}$	Substrate temperature
